# Genomic data integration systematically biases interactome mapping

**DOI:** 10.1371/journal.pcbi.1006474

**Published:** 2018-10-17

**Authors:** Michael A. Skinnider, R. Greg Stacey, Leonard J. Foster

**Affiliations:** 1 Michael Smith Laboratories, University of British Columbia, Vancouver, Canada; 2 Department of Biochemistry, University of British Columbia, Vancouver, Canada; University of California, San Diego, UNITED STATES

## Abstract

Elucidating the complete network of protein-protein interactions, or interactome, is a fundamental goal of the post-genomic era, yet existing interactome maps are far from complete. To increase the throughput and resolution of interactome mapping, methods for protein-protein interaction discovery by co-migration have been introduced. However, accurate identification of interacting protein pairs within the resulting large-scale proteomic datasets is challenging. Consequently, most computational pipelines for co-migration data analysis incorporate external genomic datasets to distinguish interacting from non-interacting protein pairs. The effect of this procedure on interactome mapping is poorly understood. Here, we conduct a rigorous analysis of genomic data integration for interactome recovery across a large number of co-migration datasets, spanning diverse experimental and computational methods. We find that genomic data integration leads to an increase in the functional coherence of the resulting interactome maps, but this comes at the expense of a decrease in power to discover novel interactions. Importantly, putative novel interactions predicted by genomic data integration are no more likely to later be experimentally discovered than those predicted from co-migration data alone. Our results reveal a widespread and unappreciated limitation in a methodology that has been widely used to map the interactome of humans and model organisms.

## Author summary

Proteins interact to perform normal cellular functions, and disruption of these interactions results in many human diseases. Consequently, defining the complete map of protein-protein interactions in humans and other organisms is of great interest. Mass spectrometry has been widely used to map protein interaction networks, but identification of true protein-protein interactions within the resulting massive proteomic datasets is challenging. One widely used approach integrates proteomic data with publicly available functional genomics data, such as mRNA coexpression or protein coevolution, to define likely interactions. Here we report that this technique, which we refer to as genomic data integration, introduces systematic biases into the resulting protein interaction networks. Genomic data integration increases the functional coherence of these networks, but at the expense of decreasing power to discover novel interactions, increasing bias towards highly-studied proteins, and impairing recognition of protein complexes. Furthermore, interactions predicted from proteomic data alone are no less likely to later be discovered. Our results indicate that a widely used computational protocol limits discovery of novel protein-protein interactions, with implications for attempts to define the molecular basis of human physiology and disease.

## Introduction

Biological functions are mediated by the dynamic organization of proteins and other biomolecules, including DNA, RNA, and metabolites, into complex networks of interactions [1]. Perturbations of these networks are implicated in human disease [2]. Consequently, efforts to chart the network of biologically relevant protein-protein interactions (the “interactome”) occupy a central position in the endeavour to understand the biochemical basis of human physiology and disease pathobiology [3,4]. Nearly two decades of study have produced initial systematic interactome maps of humans [5–9] and model organisms. However, traditional methods for interactome mapping, such as yeast two-hybrid (Y2H) or affinity purification-mass spectrometry (AP-MS) require the introduction of tags into all proteins of interest in order to provide a measurable readout [10,11]. Such tags are laborious to introduce, and may disrupt the native interactions or localization of the protein [12]. Furthermore, these methods cannot easily be applied to identify temporal rearrangements in the interactome, instead yielding static pictures of the cellular protein interaction network [13,14].

In response to interest in assembling complete maps of human and model organism interactomes, and in identifying changes in protein-protein interactions in response to perturbation, a number of experimental techniques have emerged to increase the throughput and resolution of interactome mapping using co-migration, also referred to as protein correlation profiling (PCP). Recently, we described an approach that combines PCP with stable isotope labeling by amino acids in cell culture (PCP-SILAC) and size exclusion chromatography (SEC), and applied this method to identify rearrangements in the interactome of HeLa cells following stimulation with epidermal growth factor (EGF) [13]. More extensive fractionation methods have also been employed to identify co-migrating proteins across a wide range of biochemical conditions [9,15]. Importantly, although neither SEC-PCP-SILAC nor orthogonal co-migration approaches yield direct evidence of physical protein-protein interactions, they provide a basis for inference of co-complex membership based on correlated protein abundance across conditions designed to separate protein complexes based on their size or other biochemical properties.

Co-migration methods for interactome mapping quantify thousands of proteins across a large number of fractions. Discriminating interacting from non-interacting protein pairs within the resulting complex and noisy proteomic datasets represents a significant computational challenge. Consequently, a number of published computational pipelines incorporate additional sources of evidence supporting the presence or absence of a physical interaction, derived from external genomic datasets, in machine-learning classifiers. Diverse sources of publicly available functional genomics data supporting functional or physical association have been incorporated into published classifiers: for instance, mRNA co-expression, protein co-evolution, or gene co-citation in published literature abstracts. Published protein-protein interactions, either from previous high-throughput studies or compiled from small-scale experiments, may also be incorporated as sources of evidence, as may interactions between orthologous proteins in other model organisms (“interologs”) [16].

The popularity of incorporating external genomics datasets in co-migration data analysis attests to the widespread belief that this methodology increases the quality of the resulting interaction networks, by enabling the classifier to more accurately discriminate between true and spurious interactions. Consequently, this strategy has been employed by several large-scale interactome mapping efforts, e.g. [9,15,17,18]. However, despite its broad use, the effects of genomic data integration on the global properties of the protein-protein interaction networks recovered from proteomic data has not been rigorously assessed. A major goal of large-scale interactome mapping projects is to discover novel interactions, yet it seems intuitively likely that incorporating information about known interactions, or functional associations, could decrease the power of a classifier to reveal truly novel interactions within experimental datasets. Moreover, publicly available genomics datasets are often biased towards well-studied proteins, and it is unclear whether this bias is propagated into the composition and topology of the resulting interaction networks. Finally, the precise effects of each external functional genomics data type have not been rigorously documented, and individual datasets have been integrated in a largely *ad hoc* manner. The question of which individual datasets should be integrated to optimize network quality therefore remains open. Although co-migration methods can significantly increase the throughput of interactome mapping, they nevertheless require substantial investment of time and resources to generate. It is therefore critical to ensure that the resulting datasets are analyzed in a manner that balances power to discover novel interactions with the desire to prioritize true positives for further experimental validation.

In the present study, we rigorously evaluate the effects of incorporating external genomic datasets on the quality, topology, and novelty of protein-protein interaction networks recovered from mass spectrometric data. We first apply our framework to analyze co-migration datasets we have produced using SEC-PCP-SILAC. As a baseline, we predict interactions using PrInCE [19], a naive Bayes classifier trained exclusively on dataset-derived features: e.g., the Euclidean distance or Pearson correlation between two chromatograms. Our intent is not to argue that our own experimental and computational pipelines represent universally optimal techniques for detecting protein-protein interactions using the principle of co-migration. Rather, we believe that our methods are sufficiently representative that our conclusions generalize more broadly to other experimental and computational approaches. In support of this argument, we present evidence that our results are qualitatively unchanged when the naive Bayes classifier used to predict interactions in PrInCE is replaced by a support vector machine, or when training classifiers on an alternative selection of data-derived features. In addition, we extend our analysis to recently published co-migration datasets generated by others, using orthogonal experimental methods, to demonstrate that our results apply to data analysis of co-migration experiments in general. We find that, while incorporating external genomic datasets increases the power of the resulting networks to predict protein function, it leads to a substantial decrease in the proportion of novel interactions discovered. Because novel interactions could represent either true undetected interactions or false positive associations, we apply a time-split cross-validation approach [20] to estimate the proportion of true positives among putative novel interactions. Importantly, we find that novel interactions predicted with or without external genomic datasets are equally likely to be discovered by subsequent studies, suggesting that genomic data integration impedes discovery of novel interactions between proteins without previously known functional associations. Our results reveal a widespread and unappreciated limitation in a methodology used to process proteomic datasets, with implications for efforts to map the human interactome.

## Results

**Fig 1:**
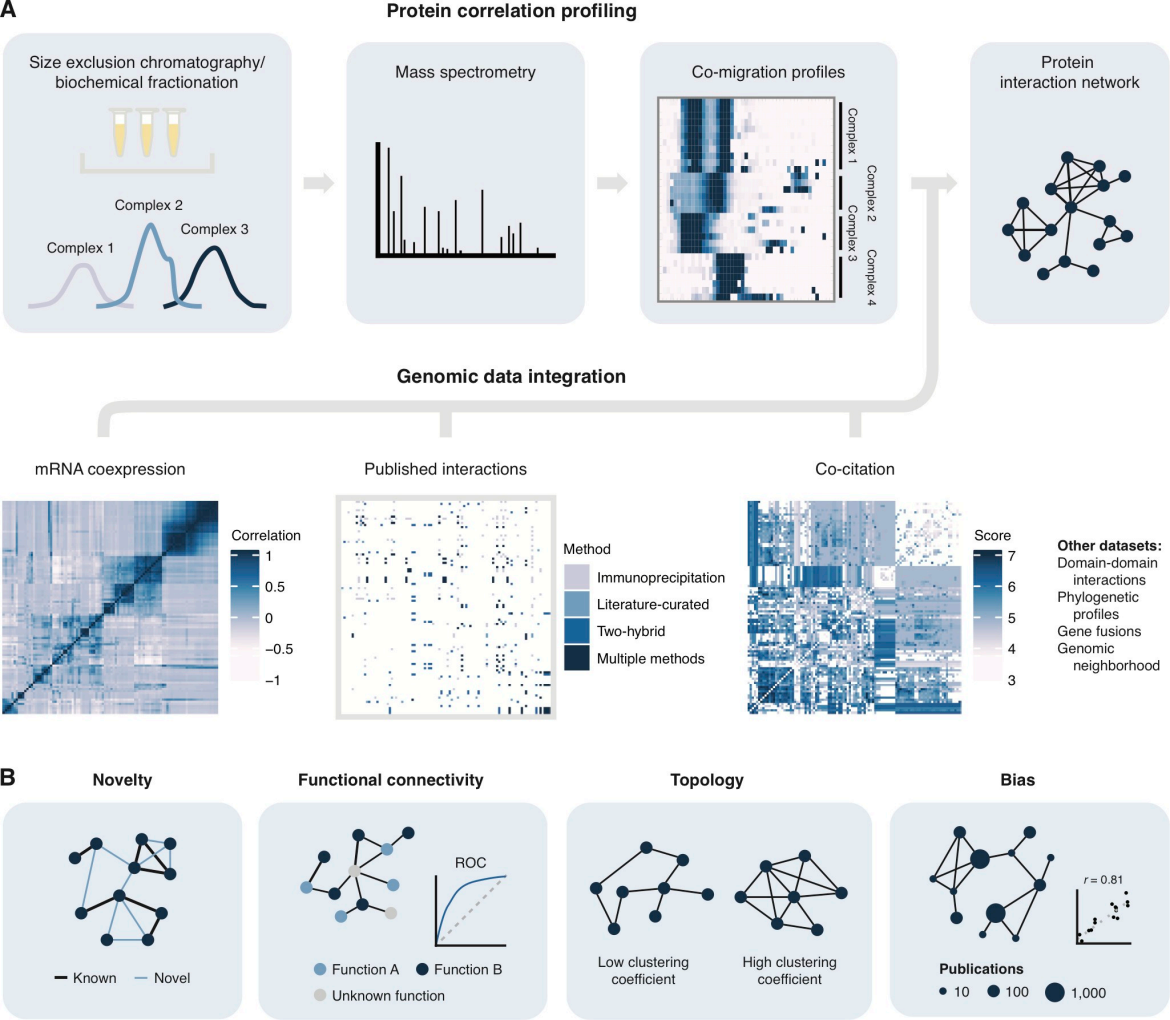
Genomic data integration for interactome mapping. (A) Overview of co-migration workflow for interactome mapping and integration of external genomic datasets. (B) Primary outcomes analyzed in the present study.

Published computational pipelines for analysis of co-migration datasets make use of variable numbers and types of external genomic datasets to identify interactions. We sought to systematically evaluate the effects of genomic data integration on the properties of the interactomes recovered from raw proteomics data. The framework for our study was, therefore, as follows: We first integrated variable numbers of external genomic datasets into machine-learning classifiers and used each classifier to predict interactions from 16 co-migration datasets, generated using SEC-PCP-SILAC, SEC coupled to label-free quantification (SEC-LFQ), or biochemical co-fractionation. We then analyzed the following properties for each network: (i) its biological coherence, defined in the following section; (ii) the proportion of interactions within the network that were novel; (iii) the clustering coefficient of the network, a measure of its ability to recover fully connected protein complexes; and (iv) the degree of bias within the network towards highly studied proteins. While none of these outcomes should be taken as a simple indicator of network quality in isolation, in combination they capture relevant properties of the analytical approach used to recover interaction networks from co-migration data. Next, we decomposed the effect of individual genomic features by adding external datasets one at a time and evaluated the same properties of the resulting networks. In addition, since functional genomics datasets exhibit varying degrees of incompleteness, we evaluated the robustness of the recovered networks to variations in the completeness of the training data. Finally, we confirmed our results were robust to the choice of classifier. An overview of our study design is provided in Fig 1.

We began by analyzing the functional connectivity of each network, defined as the degree to which the function of any given protein can be predicted from those of its interacting partners, based on the principle of ‘guilt by association’ [21,22]. In this analysis, we labeled each protein with its annotated Gene Ontology (GO) terms, then withheld a subset of those labels. We then asked how accurately these withheld GO terms can be predicted on the basis of the interaction network alone, by assigning a score to each protein-GO term pair that reflects the proportion of the protein’s interacting partners that are annotated with the same term. This procedure was repeated three times, and the mean area under the receiver operating characteristic (ROC) curve (AUROC) was calculated for each GO term. The resulting distribution of AUROCs provides a quantitative overview of the biological coherence of the network, with higher AUROCs characteristic of a network in which proteins with a given function tend to be connected to other proteins with the same function. Importantly, this measure of a network’s biological coherence is directly aligned with a key task for which protein-protein interaction (PPI) networks have been used (that is, protein function prediction) [23,24].

**Fig 2:**
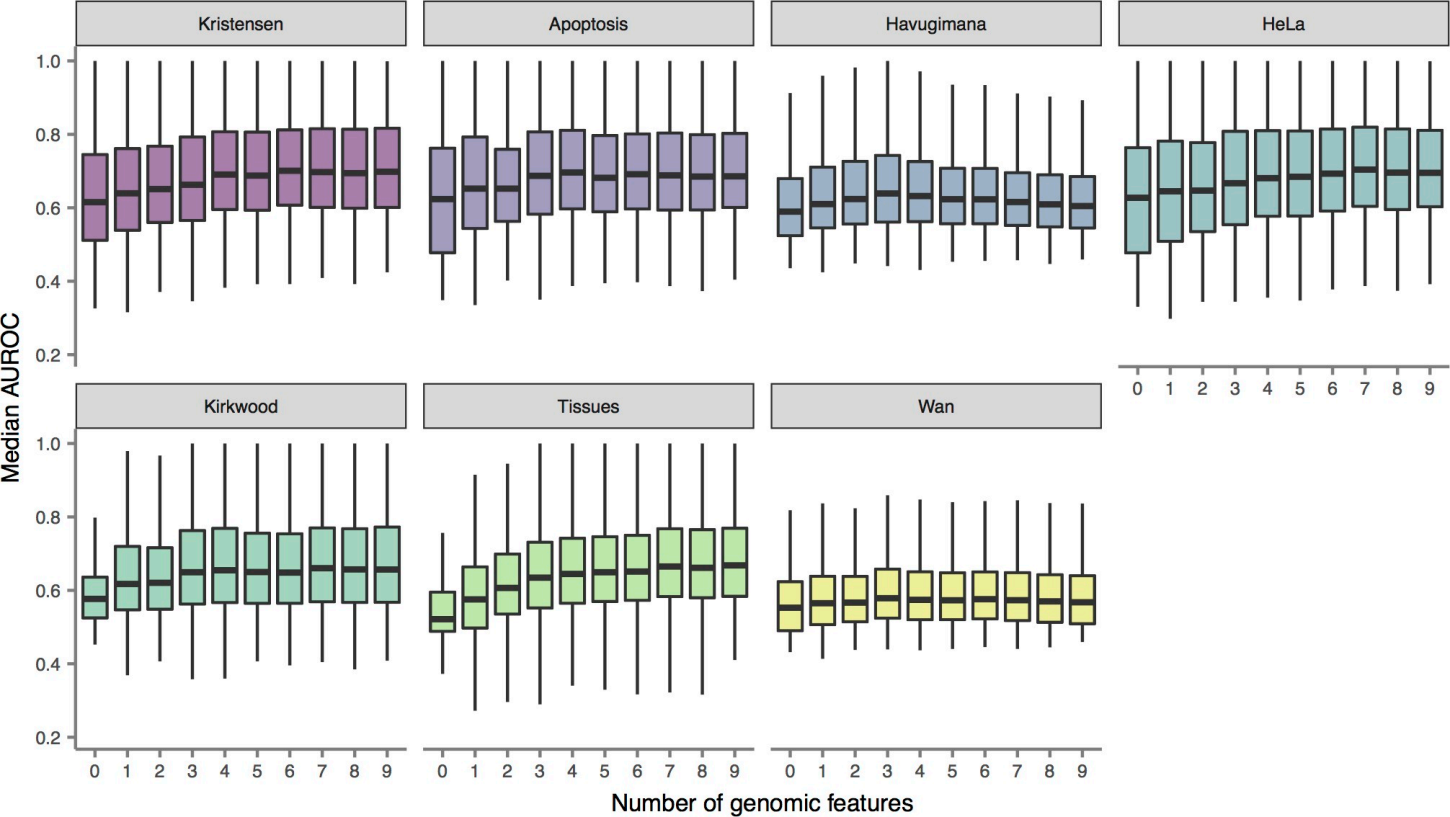
Functional connectivity of co-migration interactomes. PPI networks were predicted from co-migration data using machine-learning classifiers trained on raw co-migration data alone, or co-migration data supplemented with combinations of one to nine external genomic features. For each network, the functional connectivity was calculated as the distribution of AUROCs for prediction of protein function by neighbor voting in three-fold cross-validation.

We predicted protein interaction networks by supplementing dataset-derived features with combinations of zero to nine external genomic features (Methods) and calculated the median AUROC for each network across all GO terms. The number of genomic features used to train each classifier was strongly and significantly correlated with the functional connectivity of the resulting networks (Fig 2; Spearman’s ρ = 0.40, P = 3.2 × 10^−51^). This conclusion held for both datasets generated using SEC-PCP-SILAC (ρ = 0.49, P = 5.5 × 10^−63^), as well as SEC-LFQ datasets (ρ = 0.37, P = 6.6 × 10^−3^); no significant correlation was observed for the biochemical co-fractionation datasets (ρ = 0.02, P = 0.81). Thus, using conventional measures of a network’s biological coherence, interaction networks constructed using genomic data integration outperform those constructed using co-migration data alone.

**Fig 3:**
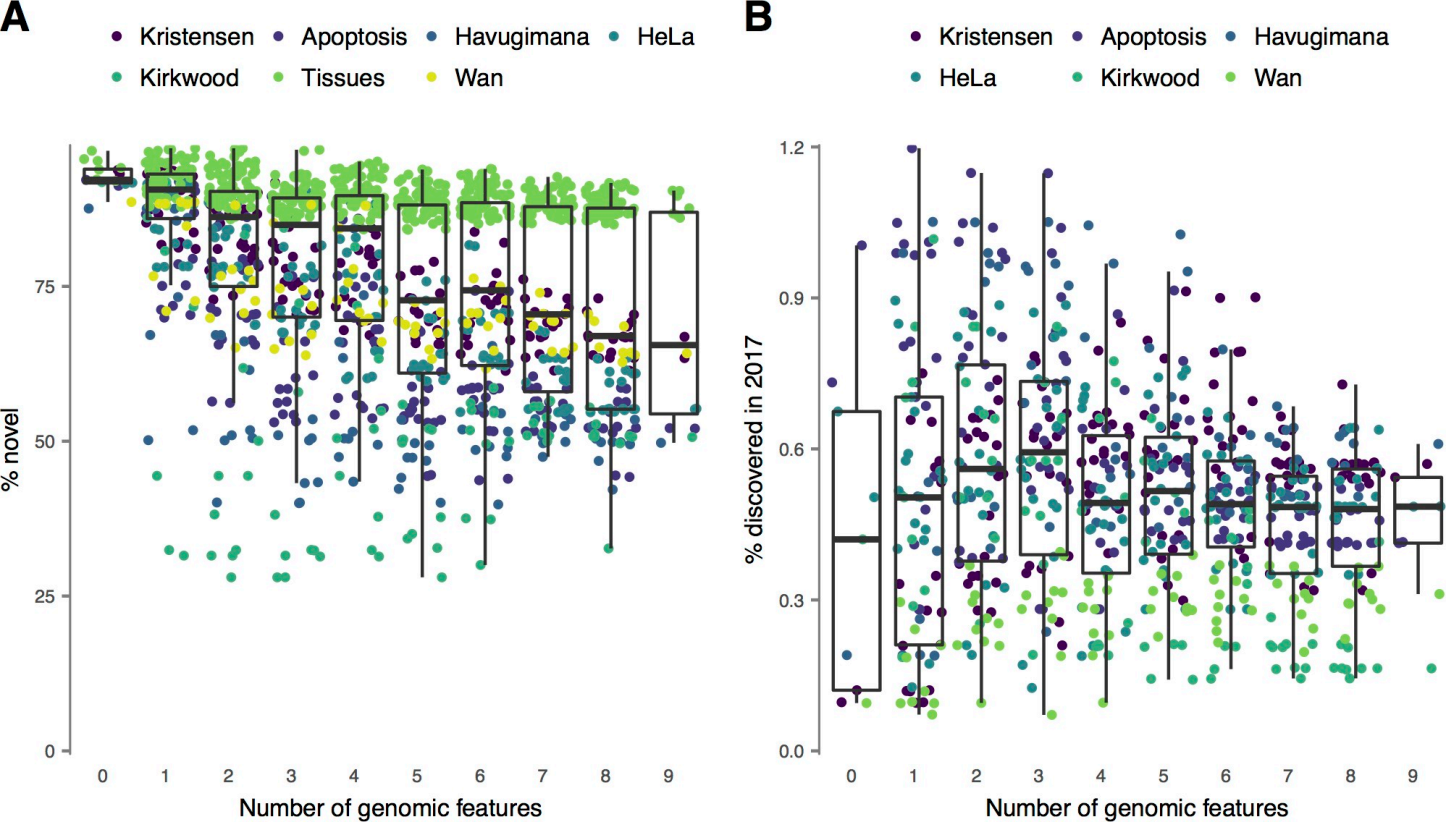
Novel interactions in co-migration interactomes. (A) Comprehensive databases of human and mouse protein-protein interactions were compiled and used to calculate the proportion of novel interactions within interaction networks recovered from co-migration data alone or supplemented with combinations of one to nine external genomic datasets. Each point represents a network derived from a machine-learning classifier incorporating a different combination of external genomic features. (B) Interactions discovered in 2017 only were withheld from the database of known interactions and used to estimate the proportion of true positives among putative novel interactions by time-split cross-validation. The ‘Tissues’ dataset is omitted as insufficient interactions were available to conduct time-split cross-validation in mouse.

Although genomic data integration increased the accuracy of protein function prediction from interactome topology, incorporating information about known functional associations or physical interactions could decrease the power of a classifier to discover novel interactions. Given that a primary goal of high-throughput interactome mapping projects is to discover novel interactions, we therefore undertook a systematic effort to compile known PPIs across seventeen databases (Methods) and used these catalogs of known interactions to calculate the proportion of previously known interactions in each network generated from co-migration data. We observed a strong negative correlation between the number of external genomic datasets integrated into the classifier and the proportion of novel interactions within the network (Fig 3A; Spearman’s ρ = –0.34, P = 1.9 × 10^−36^). This trend remained significant for both SEC-PCP-SILAC (ρ = –0.38, P = 4.0 × 10^−36^) and biochemical co-fractionation datasets (ρ = –0.43, P = 1.5 × 10^−8^), although not the SEC-LFQ dataset (ρ = 0.014, P = 0.90). These observations suggest that the increased functional connectivity of networks recovered by integrating external genomic datasets could come at the expense of decreasing power to discover novel interactions.

Putative novel interactions could represent truly undiscovered interactions within an experimental dataset, but they could also reflect spurious or noisy associations within the data. Thus, a critical outcome in evaluating genomic data integration is what fraction of putative novel interactions actually represent true positives. To estimate this proportion, we performed a time-split cross-validation experiment [20]. We withheld interactions reported only in 2017 (n = 34,237) when identifying known interactions, then calculated the proportion of putatively novel interactions that were subsequently identified in 2017 within the human co-migration datasets. If putative novel interactions recovered by genomic data integration are more likely to represent true positives, then this procedure should be associated with a higher rate of interaction discovery in the validation set. However, surprisingly, we observed the inverse relationship, with a moderate *negative* correlation between genomic data integration and the likelihood of a putatively novel interaction later being discovered (Fig 3B; Spearman’s ρ = –0.13, P = 5.6 × 10^−3^). We confirmed this moderate negative trend for SEC-PCP-SILAC (ρ = –0.18, P = 1.1 × 10^−4^) and SEC-LFQ (ρ = –0.64, P = 1.2 × 10^−10^) datasets individually, but found the reverse trend for the biochemical co-fractionation datasets (ρ = 0.21, P = 0.0066). However, in aggregate, the absence of a positive effect of genomic data integration on the proportion of putative novel interactions that were subsequently validated suggests that the effect of this procedure on the biological relevance of the recovered interactions is negligible. We therefore conclude that genomic data integration decreases power to discover true, condition-specific interactions between proteins that are not yet linked by a known functional association. To ensure our results were insensitive to the temporal cutoff used in our time-split cross-validation scheme, we repeated these analyses by withholding interactions discovered in 2016 or later. This analysis confirmed the absence of a positive trend (ρ = –0.057, P = 0.13; S1 Fig). Finally, we confirmed that our results were robust to controlling networks based on the false discovery rate, rather than number of interactions (S2 Fig), finding that genomic data integration decreased the proportion of novel interactions within these networks (ρ = –0.31, P = 6.4 × 10^−22^), but did not have a significant effect on the proportion of interactions that were subsequently discovered under either time split (ρ = –0.039, P = 0.45 and ρ = –0.029, P = 0.57 for 2017 and 2016, respectively).

We note as a caveat to this conclusion that, when we analyzed the subset of networks presented in Fig 3B that were generated with combinations of zero and three features, there was a marginally significant correlation between the number of external genomic features integrated and the proportion of interactions that were subsequently discovered (ρ = 0.17, P = 0.0055). This result might be interpreted to suggest that a limited degree of genomic data integration can have a positive effect on the quality of the recovered interaction network. However, this post-hoc subgroup analysis should be interpreted cautiously, particularly since the correlation was no longer statistically significant when analyzing networks generated with combinations of up to four features (ρ = 0.028, P = 0.59). We additionally asked whether any specific combinations of external genomic datasets consistently had a positive effect on the proportion of putative novel interactions that were subsequently discovered, relative to baseline networks recovered without external genomic datasets, but found that no combination had a significant effect after multiple hypothesis testing correction (all P > 0.05).

**Fig 4:**
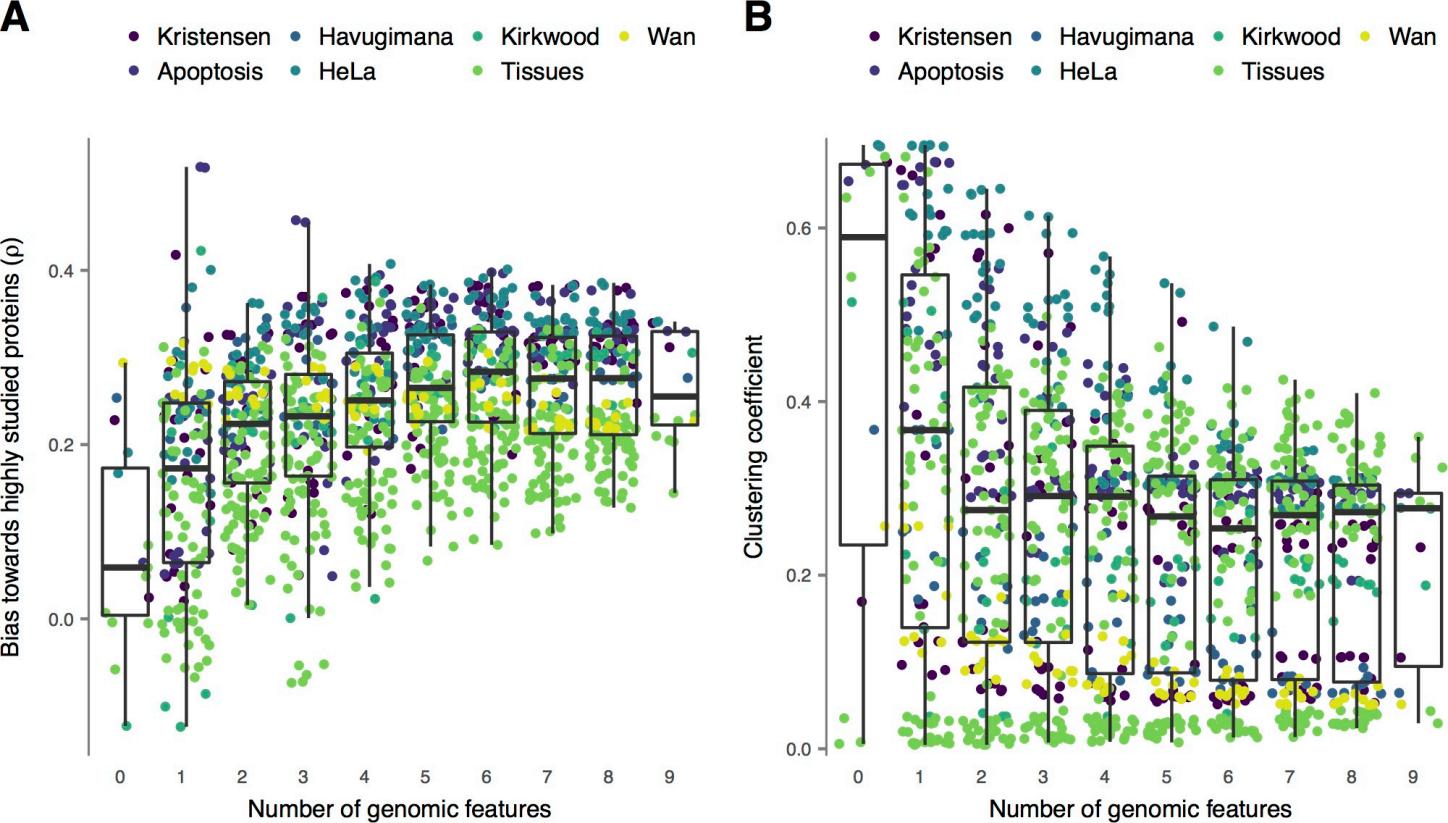
Bias towards highly studied proteins and protein complex recovery in co-migration interactomes. (A) Correlation between protein degree (number of interacting partners) and number of publications describing that protein in interaction networks recovered from co-migration data alone or supplemented with combinations of one to nine external genomic datasets. (B) Global clustering coefficients of protein-protein interaction networks recovered from co-migration data alone or supplemented with combinations of one to nine external genomic datasets.

We next considered another potential bias introduced by genomic data integration: in particular, many genomic datasets are characterized by bias toward highly studied proteins. For example, literature-curated interactions from small-scale studies disproportionately involve well-studied proteins [8]. Similarly, interacting protein domains require three-dimensional structural templates to define, and therefore introduce bias towards proteins that have been studied using the techniques of structural biology. Incorporating these biased datasets into computational pipelines to analyze co-migration data introduces the possibility that biases towards highly studied proteins will be propagated into the resulting interaction networks. Such biases have been described in the context of a large ‘uncharted zone’ in interactomes derived solely from small-scale, literature-curated experiments, wherein the products of many human disease genes associate with few or no interacting partners, while a smaller number of highly studied proteins are densely connected [8]. We developed a quantitative metric of this bias within each co-migration interactome by calculating the Spearman correlation between the number of interactions predicted for each protein, and the number of publications in which it has been mentioned. Networks derived from co-migration data alone displayed minimal bias towards highly studied proteins (median Spearman’s ρ = 0.059), but the degree of bias increased sharply with the number of external genomic datasets incorporated (Fig 4A; Spearman’s ρ = 0.32, P = 8.3 × 10^−32^), a correlation that remained significant across two of three experimental methods (SEC-PCP-SILAC, ρ = 0.36, P = 1.9 × 10^−32^; SEC-LFQ, ρ = 0.56, P = 6.1 × 10^−8^; biochemical co-fractionation, ρ = –0.10, P = 0.19). Thus, in addition to containing a lower proportion of novel interactions overall, interaction networks predicted using genomic data integration display a greater bias towards highly studied proteins, whose functions are more likely to already be well understood.

Many proteins assemble into not only pairwise interactions, but higher-order, multi-protein complexes. In graph theoretic terms, these complexes can be described as cliques, as every protein in the complex co-migrates with every other protein in the complex. A global topological measure that reflects the tendency of interacting proteins within a network to form cliques, and therefore complexes, would thus provide an orthogonal, high-level assessment of the biological relevance of the network, given that the aim of all the co-migration experiments analyzed here was identify protein complexes co-migrating across one or more separation gradients. Such a measure is provided by the clustering coefficient of a network, which measures the probability that any two proteins connected to a given third protein are themselves connected in the interaction network [1]; a network with a high clustering coefficient is thus one with a global topology characteristic of protein complexes. We calculated the clustering coefficients for each network as a measure of the tendency of the classifier to preferentially identify protein complexes, and found that, although clustering coefficients varied considerably across datasets, genomic data integration was consistently associated with decreased clustering coefficients across all three methods (Fig 4B; Spearman’s ρ = –0.20, P = 1.2 × 10^−12^; SEC-PCP-SILAC, ρ = –0.23, P = 7.7 × 10^−14^; SEC-LFQ, ρ = –0.14, P = 0.22; biochemical co-fractionation, ρ = –0.77, P = 5.3 × 10^−33^). Importantly, the clustering coefficients of the resulting interaction networks reflect an outcome independent of investigator biases inherent to the Gene Ontology and PPI databases. Our analysis of interaction network topology therefore suggests that genomic data integration impedes the identification of co-eluting complexes within co-migration datasets.

In all analyses described above, we made use of a naive Bayes classifier to distinguish interacting and non-interacting protein pairs. However, published computational pipelines have employed a variety of machine-learning methods. To confirm that our results were insensitive to the precise design of the computational pipeline, we repeated these experiments using support vector machines (SVMs) to classify interactions. We found our results were qualitatively unchanged (S3 Fig), indicating our conclusions are robust to the exact statistical techniques used to integrate known functional associations with co-migration data to map interactions.

**Fig 5:**
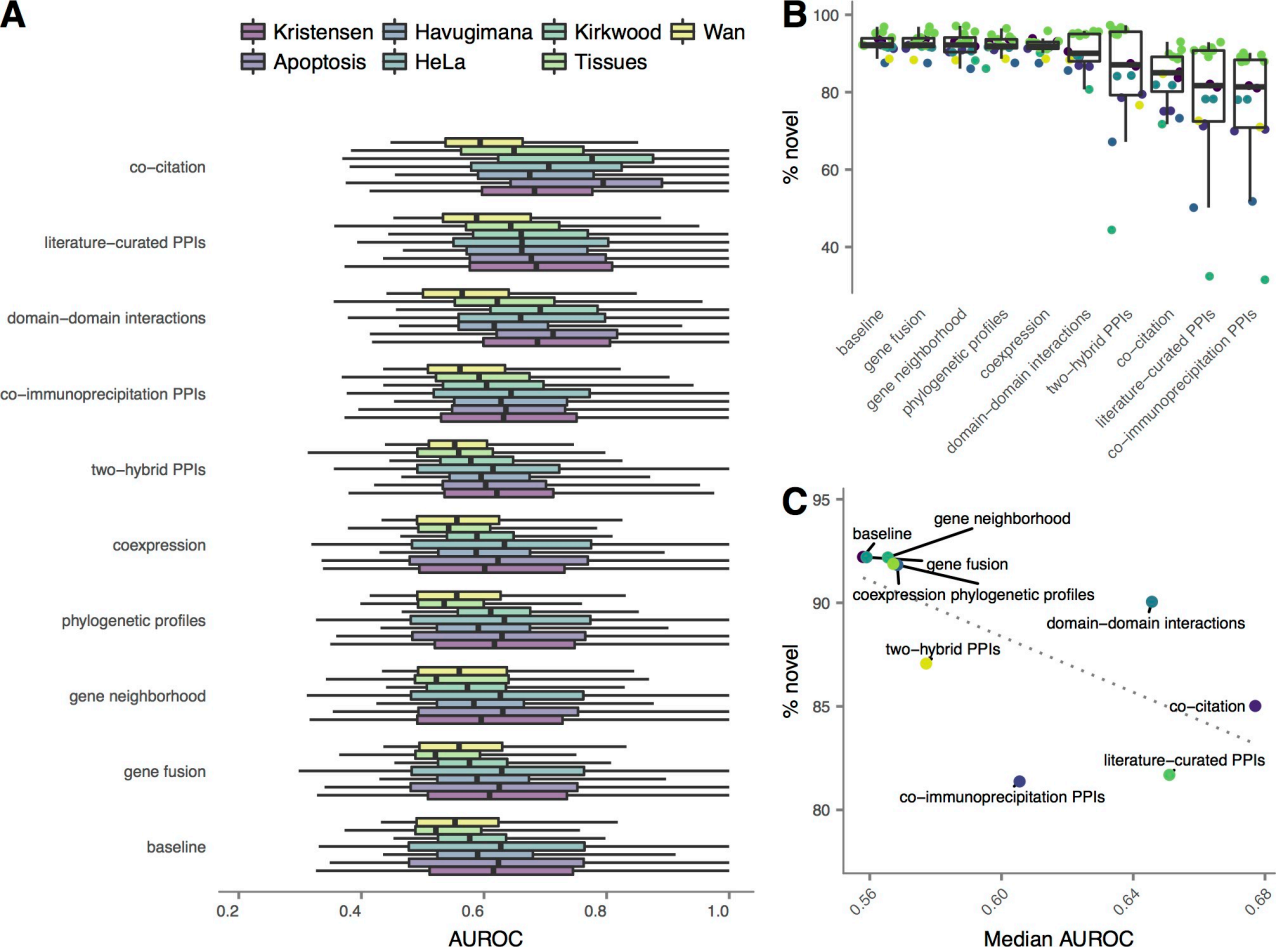
Effect of individual genomic features on functional connectivity and novelty in co-migration interactomes. (A) Functional connectivity and (B) proportion of novel interaction networks recovered from co-migration data alone, or in combination with individual external genomic features. (C) Median proportion of novel interactions vs. median functional connectivity of networks recovered in combination with individual genomic features. Dotted line shows ordinary least squares linear regression.

Our results thus far indicate that, when predicting interaction networks from co-migration data, increasing genomic data integration is associated with increased functional connectivity, at the expense of decreasing power to discover novel interactions or reveal complete protein complexes. A remaining question is whether the trends described here apply to all genomic datasets, or whether individual datasets can be isolated as drivers of improved functional connectivity or decreased novelty. A single feature capable of increasing the biological coherence of the resulting networks, while leaving the proportion of novel interactions largely unchanged, would be highly desirable for co-migration data analysis. To address this question, we incorporated individual genomic features into classifiers, and evaluated the functional connectivity and novelty of the resulting networks. Eight of the nine features tested here resulted in a significant increase in functional connectivity relative to a baseline of exclusively dataset-derived features (Fig 5A; all P ≤ 1.6 × 10^−12^, Fisher’s method), with the sole exception being gene fusion (P > 0.99). However, the same eight features resulted in highly significant increases in the proportion of previously known interactions (Fig 5B; all P ≤ 7.0 × 10^−26^, Fisher’s method, except P > 0.99 for gene fusion). The magnitude of these effects were variable, with the largest decreases in the proportion of novel interactions observed upon integration of co-citation data or previously published interactions. In contrast, smaller changes were observed for mRNA coexpression and phylogenetic profiles, suggesting these constitute less biased sources of genome-wide functional associations (Fig 5C).

We also analyzed the effect of individual features on network bias towards highly studied proteins, network clustering coefficients, and the proportion of putatively novel interactions later discovered in time-split cross-validation (S4 Fig). Notably, while none of coexpression, phylogenetic profiles, gene fusion, or gene neighborhood had a statistically significant impact on network bias towards highly studied proteins (S4A Fig; all P ≥ 0.64, Brunner–Munzel test), all sources of interologous PPIs increased bias towards well-studied proteins (all P ≤ 5.7 × 10^−3^), as did domain-domain interactions (P = 5.5 × 10^−4^); as expected, literature co-citation had the greatest effect (P = 2.8 × 10^−9^). In contrast, no individual feature had a significant impact on the clustering coefficient of the network (S4B Fig; all P ≥ 0.051, Brunner–Munzel test). This finding suggests that integration of several features is required to significantly impact protein complex recovery from co-migration data. Finally, we analyzed the proportion of putative novel interactions that were subsequently discovered in our time-split cross-validation scheme, a proxy for the proportion of putative novel interactions that correspond to true positives. Our results were identical regardless of the time point used to split the training dataset: domain-domain interactions and interologous literature-curated or co-immunoprecipitation PPIs reproducibly increased the proportion of putative novel interactions that were later discovered (all P ≤ 3.5 × 10^−4^, Fisher’s method). Surprisingly, co-citation data reproducibly led to a significant *decrease* in the proportion of true positive interactions (P ≤ 1.6 × 10^−11^); the remaining features did not have a significant effect in isolation (P ≥ 0.33). Taken together, these analyses reinforce the notion that integration of different functional genomic features has distinct and characteristic impacts on the properties of recovered interaction networks and provide additional support for the use of mRNA co-expression and phylogenetic profiles in cases where genomic data integration is felt to be necessary.

**Fig 6:**
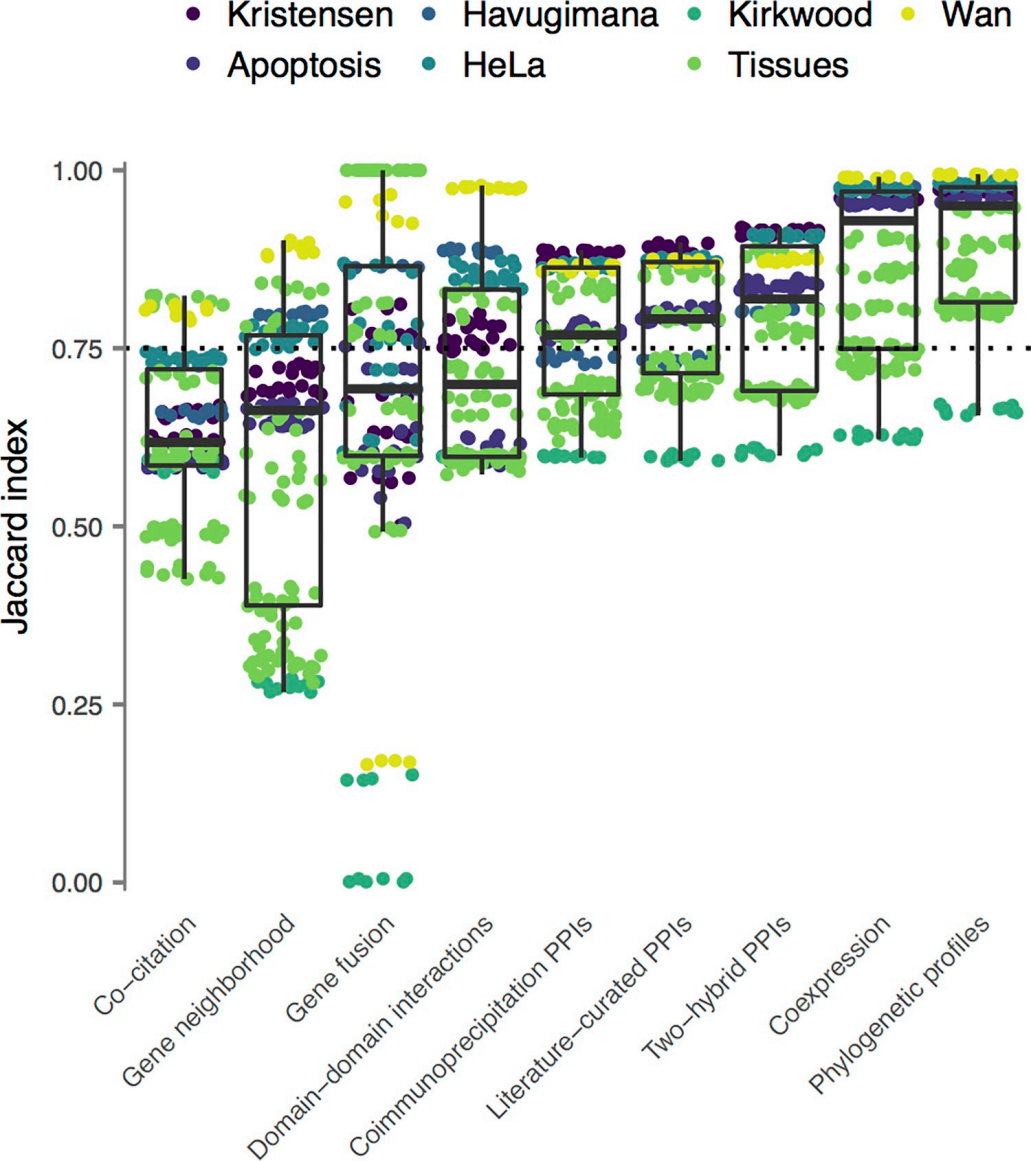
Robustness of predicted interaction networks to incomplete genomic data. The effect of incompleteness in external genomic data was analyzed by randomly withholding 20% of each genomic feature in five-fold cross-validation. The Jaccard index between networks derived from the same co-migration dataset was calculated as a measure of robustness. The dotted line corresponds to a Jaccard index of 0.75.

A final question we aimed to address is how stable co-migration interactomes are to slight perturbations in external genomic datasets. Many functional genomics datasets are incomplete: for example, the identification of domain-domain interactions relies on limited three-dimensional structural information, and existing interactome maps of model organisms are limited in scope. We simulated incompleteness of external genomic features by randomly withholding 20% of the annotations for each feature and predicted interaction networks with the resulting incomplete datasets. This procedure was repeated five times, and the Jaccard indices between all pairs of interaction networks were calculated as a measure of robustness to incomplete data. Networks displayed variable degrees of stability to perturbations in external genomic datasets, with four of nine features resulting in a median Jaccard index less than 0.75 between networks derived from the same co-migration data (Fig 6). This finding is concerning, as it suggests that integration of these features impedes robust, stable identification of interacting protein pairs. In contrast, co-expression, phylogenetic profiles, and all three sources of interologous PPIs provided comparatively greater robustness to incomplete data, suggesting these datasets yield more useful information for high-throughput interactome mapping.

## Discussion

Elucidating the complete interactome of humans and model organisms has been a longstanding goal of modern high-throughput biology, yet conventional methods to reveal PPIs are labour-intensive and not amenable to studying the interactome under physiological conditions or in response to stimulus. To address this gap, a new generation of methods for interactome mapping using co-migration have been developed and successfully applied in recent years [9,13,15,17]. However, best practices for analysis of the unique proteomic datasets generated by these methods remain poorly defined. We conducted an extensive analysis of one widely used approach, which integrates known functional associations with co-migration data to define interactomes using machine learning. Our results reveal that interaction networks recovered from raw data with this technique perform better on conventional benchmarks, such as their ability to predict protein function. However, this improved performance is associated with a decreased ability to identify novel interacting protein pairs within experimental data. Importantly, novel interactions could represent either true, undiscovered interactions, or false positive protein pairs. Our analysis suggests that classifiers trained without external data are, for the most part, discovering truly novel and potentially condition-specific interactions that lack known functional associations. In addition, we find that integration of external genomic datasets propagates bias towards highly-studied proteins into interaction networks and precludes accurate recognition of co-eluting complexes. Taken together, these findings suggest that genomic data integration biases the resulting protein interaction networks towards known functional associations, while impeding the discovery of true physical interactions between protein pairs that lack known functional associations in existing datasets.

The concept of functional association has proven tremendously useful for predicting gene function [25] and interpreting genome-wide association study data [26,27], among other genome-scale applications. However, a key point highlighted by our analysis is that functional association is not synonymous with physical interaction. Conversely, many apparently interacting proteins lack known functional associations. As we show, this distinction is critical in interpreting high-throughput efforts to map the interactome by co-migration, given that a primary goal of such projects is to discover previously unknown physical interactions. In fact, interactions substantiated solely from raw data appear no less likely than interactions substantiated by functional associations to later be discovered. This observation attests to the high quality of co-migration data. However, it also highlights the noisiness of existing functional annotations: for instance, mRNA co-expression is known to be systematically biased by the spatial proximity of gene pairs [28], while shared evolutionary trajectories can indicate participation in related functional pathways or processes rather than direct physical interaction [29]. Perhaps as a result, genomic data integration appears to decrease the power of machine-learning approaches to identify co-eluting protein complexes, as judged by a topological metric independent of investigator biases within known interaction databases or functional annotations in the Gene Ontology. Our results thus reveal that emphasis of prior functional associations comes at the expense of ability to reveal previously unknown biology. Conversely, the lack of a known functional association between pairs of putatively interacting proteins should not be used to rule out novel interactions.

Our analysis suggests that integration of external genomic datasets during the process of network construction from co-migration data impedes the discovery of novel interactions or protein complexes. However, there is clearly value in integrating genomic data after network construction, in order to validate the overall quality of the experimental network, or to compare datasets generated by different experimental methods [30]. Notably, such comparative analysis is made more difficult by the fact that many published studies have integrated publicly available functional genomics datasets with their mass spectrometric data to derive the final protein interaction networks. Consequently, these published interaction networks combine experimentally detected interactions with previously known functional associations, making it difficult to tease apart the relative contributions of each to the network. Thus, genomic data integration also represents a barrier to the comparative analysis of existing datasets.

Clearly, the scope of our analysis is not exhaustive: one can envision the addition of further external genomic datasets into classifiers designed to reconstruct interactome networks from raw co-migration proteomic data, or alternative methods of representing the features considered in this analysis (for instance, incorporating evidence of protein co-evolution at the sequence level, instead of genomic co-occurrence). However, in selecting the particular external genomic datasets that were considered within this study, our aim was to reflect those that have been incorporated in published co-migration projects, in order to ensure the conclusions of our study are relevant to the analytical protocols that have actually been implemented within the field to date. In this respect, it is noteworthy that no individual combination of external genomic datasets had a significant positive effect on the proportion of putative novel interactions that were subsequently discovered, under our time-split cross-validation scheme. Moreover, by dissecting the individual contribution of each external genomic feature to the properties of the resulting interaction networks, we provide an extensive resource to understand the individual impacts of the nine external genomic features evaluated herein.

On the basis of our analysis, we argue that bioinformatic methods for interpretation of co-migration data should rely exclusively on dataset-derived features if a primary aim of a research effort is to discover novel interactions. We believe that this recommendation is particularly relevant to efforts to map the interactome across physiological conditions or in response to perturbation. It is tempting to additionally speculate that re-analysis of published co-migration data, using solely data-derived features, has the potential to reveal previously unknown interactions and complexes [31]. If integration of additional data is deemed essential to maximize the success of labour-intensive downstream validation, only one or a few features should be included: we found that integration of even a single feature reliably resulted in a significant decrease in the proportion of novel interactions recovered. Our analysis suggests mRNA co-expression, phylogenetic profiling, and domain-domain interactions are among the most appropriate features for this purpose, as they yield significant increases in functional connectivity at the expense of relatively modest increases in the proportion of novel interactions (Fig 5C). Finally, as similar genomic data integration techniques have been employed to map the human interactome using AP-MS, our findings are likely to generalize to mass spectrometry-based efforts to map the interactome beyond co-migration.

### Conclusions

We find that a commonly used computational technique for analysis of co-migration data hinders the discovery of novel interactions from raw proteomics datasets, accentuates bias towards extensively studied proteins, and impedes accurate recovery of co-eluting complexes. Importantly, interactions predicted from raw data alone appear no less likely to later be experimentally discovered. This leads us to recommend that bioinformatics pipelines for interactome mapping from co-migration datasets should rely exclusively on this raw data, when possible. When integration of additional features is deemed essential, mRNA co-expression, phylogenetic profiling, and domain-domain interactions balance increased functional connectivity and decreased novelty. Prioritizing high-quality co-migration data over noisy functional associations will support efforts to reveal complete maps of PPIs in humans and other organisms.

## Materials and methods

### Co-migration datasets

Our initial analysis incorporated three sets of previously published co-migration experiments performed within our own laboratory using SEC coupled to PCP-SILAC (SEC-PCP-SILAC) [13,32,33]. These experiments mapped rearrangements in the interactome of HeLa cells to stimulation with EGF [13] and to infection with *Salmonella enterica* [33], as well as rearrangements in the interactome of Jurkat T cells during Fas-mediated apoptosis [32]. In addition, we analyzed a dataset mapping the interactomes of seven mouse tissues, using PCP coupled to stable isotope labelling in mammals (PCP-SILAM) [34]. Within each experiment, each condition was analyzed separately (for example, stimulated vs. unstimulated cells), for a total of 13 conditions. In addition, we analyzed a dataset generated using SEC coupled to label-free quantification (LFQ) rather than SILAC (referred to herein as “SEC-LFQ”) [35], and two datasets generated using extensive biochemical co-fractionation to separate protein complexes [9,15], in order to evaluate the robustness of our conclusions to experimental methodology. These datasets were obtained from the supplementary material of the corresponding publications, with the exception of the Wan et al. dataset, which was obtained from the supporting website (http://metazoa.med.utoronto.ca; only human experiments were analyzed here). Protein isoforms and proteins quantified in three or fewer fractions were filtered from the Kirkwood et al. data [35], leaving a total of 4,519 proteins for further analysis. Proteins quantified in three or fewer fractions, and proteins quantified in only a single experiment, were filtered from the Wan et al. data [9], and mapped to UniProt identifiers, leaving a total of 3,895 proteins for further analysis.

### External genomics datasets

We evaluated the impact of integrating nine external genomic features at the protein pair level into classifiers designed to identify protein-protein interactions (PPIs) from PCP data, including: (i) messenger RNA (mRNA) co-expression, (ii) phylogenetic profiles, (iii) domain-domain interactions, (iv) co-citation in literature abstracts, (v) gene fusion, (vi) gene proximity, and three datasets of previously known PPIs curated from (vii) small-scale experiments, (viii) two-hybrid (2H) screens, or (ix) co-immunoprecipitation (co-IP) experiments. These genomic datasets were chosen in order to mimic as closely as possible the selection of features that have been incorporated into previous genome-scale integrated functional networks [25–27], and used to train classifiers designed to identify PPIs within co-migration datasets [9,15,17,18,27]. For example, both Havugimana et al. [15] and Wan et al. [9] incorporated co-evolution, literature co-citation, mRNA co-expression, gene neighborhoods, and PPIs derived from AP/MS, Y2H, and literature curation as features in machine-learning classifiers to analyze biochemical co-fractionation datasets. Similarly, Kastritis et al. [17] incorporated gene neighborhood, gene fusion, co-expression, and literature co-occurrence to identify PPIs in a thermophilic eukaryote. Likewise, Larance et al. included high-throughput and small-scale interactions from PPI databases in their classifier to identify membrane protein complexes in a combined cross-linking and co-migration experiment [18], while Crozier et al. incorporated gene neighborhood, gene fusion, mRNA co-expression, literature co-occurrence, and experimental and literature-curated protein-protein interactions from STRING to identify protein complexes in *Trypanosoma brucei* from co-migration data [36]. We withheld functional data derived from the Gene Ontology (GO) in order to validate the functional connectivity of the resulting interaction networks, as detailed further below.

RNA expression data from healthy human and mouse tissues was obtained from the Bgee database [37], and coexpression was calculated from normalized (FPKM) expression values using the Pearson correlation coefficient. Phylogenetic profiles were constructed by mapping human or mouse proteins to 272 other species using InParanoid [38], and calculating the Pearson correlation coefficient between binarized presence/absence values [39]. Interacting domains identified within three-dimensional structure data were obtained from the 3did database [40], and Pfam domain annotations for the human and mouse proteomes were obtained from the UniProt web server to identify protein pairs possessing known domain-domain interactions [41]. Co-citation, gene fusion, and gene proximity scores were obtained from STRING, version 10.5 [42].

To assemble comprehensive datasets of previously published interactions, we systematically compiled interactions from sixteen databases: BIND [43], BioGRID [44], DIP [45], HINT [46], HIPPIE [47], HPRD [48], IID [49], InBioMap [50], MatrixDB [51], Mentha [52], MINT [53], MPPI [54], NetPath [55], PINA [56], Reactome [57], and WikiPathways [58]. From pathway databases, only the subset of information cataloguing physical PPIs was retained. When available, experimental methods supporting each interaction were recorded using the Molecular Interactions ontology [59]. Two-hybrid interactions were selected as the subset of interactions supported by the MI evidence codes containing the string ‘two hybrid’, while co-IP interactions were selected as the subset of interactions supported by evidence codes containing the string ‘coimmunoprecipitation’. In addition, all PubMed identifiers (PMIDs) supporting each interaction were recorded and used to link the interaction to all the year(s) in which it was detected using XML files distributed by PubMed. All gene and protein identifiers were mapped to UniProt accessions using identifier mapping files distributed by UniProt [41]. We assembled comprehensive databases of known interactions from human, mouse, worm, fly, and yeast using this procedure, mapping orthologs with InParanoid [38].

We used the resulting comprehensive catalog of interactions in three ways. First, we used interactions between orthologous protein pairs, detected by either (i) literature curation of small-scale experiments (LC), (ii) yeast two-hybrid (Y2H), or (iii) co-immunoprecipitation (IP) as external genomic features, integrating them into machine-learning classifiers to recover protein interaction networks from co-migration data. We defined small-scale experiments as publications reporting 25 or fewer PPIs. To minimize circularity in interaction prediction, in all cases we excluded interactions from the species of interest (i.e., human or mouse) when training classifiers on co-migration data, including only interologs detected in different species.

Second, we used all published interactions from the species of interest (i.e., human or mouse) to calculate the proportion of interactions in each recovered network that were known (i.e., previously reported in any database), defining the remaining interactions as putatively novel. In this experiment, we used two different datasets for two time-split cross-validation experiments, excluding interactions detected prior to 2017 or prior to 2016, respectively, to calculate the total proportion of previously known interactions.

Third, we used the withheld sets of interactions to estimate the proportion of putatively novel interactions that actually represent true positives by performing a time-split cross-validation [20]. Our rationale in selecting this cross-validation scheme was to provide a reasonable simulation of prospective interactome mapping projects, in which investigators have access to all previously published interactions and are interested in the proportion of predicted interactions that represent true positives. This technique was pioneered in the field of cheminformatics, where it was shown to more accurately reflect the accuracy of predictions in a prospective setting [20,60]. However, time-split cross-validation generalizes to any dataset for which each observation is time-stamped, and can reduce the impact of literature contamination or other unanticipated biases in the training dataset, thereby providing a more accurate estimation of true accuracy in comparison to the overly optimistic approach of random-split cross-validation [20]. We used two different datasets to conduct two different time-split cross-validation experiments, splitting our dataset of known interactions at those discovered in 2017 or later and in 2016 or later, respectively.

### Protein interaction prediction from co-migration data

We first predicted protein interactions using our own computational pipeline, PrInCE [19], a naive Bayes classifier that learns exclusively from dataset-derived features. By default, PrInCE incorporates six features, including the Euclidean distance between protein profiles; the Pearson correlation coefficient between profiles and its P-value; the Pearson correlation coefficient calculated from ‘cleaned’ profiles, with missing values replaced by Gaussian noise and single missing values imputed; and the distance in fractions between the maximum values of each profile. PrInCE also attempts to deconvolve each profile into a mixture of one to five Gaussians and includes the minimum Euclidean distance between any pair of Gaussians from the two profiles as a sixth feature. These features were used as input to classifiers designed to handle data from our own SEC-PCP-SILAC experiments, as well as SEC coupled to spectral counts (SEC-LFQ) [35]. In our analysis of the co-fractionation datasets, we emulated the feature selection of Havugimana *et al*. [15], calculating weighted cross-correlation and noise model correlation for each fractionation experiment, as well as a co-apex score across all fractionation experiments, as previously described. The Euclidean distance between MS1 intensity profiles was also included as a feature when analyzing the Wan et al. dataset, to emulate the authors’ original analysis [9].

In all cases, the selected features were used as input to a naive Bayes classifier, optionally supplemented with combinations of one or more features derived from the external genomic datasets described above. We evaluated all possible combinations of zero to nine external genomic features for each co-migration dataset in turn, up to a limit of ten randomly selected combinations for each number of external genomic features. The naive Bayes classifier was trained on the CORUM database [61] using ten-fold cross-validation, taking intra-complex interactions as true positives and inter-complex interactions as true negatives. Protein pairs were ranked by their median classifier scores across all ten folds, and the top 10,000 pairs were selected to form a predicted interaction network for each experiment, in order to control for the effect of interactome size on our conclusions. We additionally generated networks by controlling the false discovery rate, filtering networks with fewer than 2,000 interactions to minimize spurious associations caused by very small networks. In addition, we confirmed that our results were qualitatively unchanged when using alternative classifiers, including the random forests (RFs) implementation in the R package ‘ranger’, and the support vector machines (SVMs) implementation in the R package ‘LiblineaR’. Due to increased computational demands, only five-fold cross-validation was used for SVM and RF classifiers.

### Functional connectivity

Gene Ontology (GO) terms annotated to each protein were retrieved from the UniProt-GOA database [62]. GO annotations supported only by evidence codes ND, IPI, IEA, and/or NAS were filtered. We used the ‘EGAD’ R package [63] to perform all functional connectivity calculations in three-fold cross-validation, considering only GO terms annotated to between 0.5% and 5% of the proteins in the resulting network in order to exclude very broad or specific terms.

### Interaction novelty

We calculated the proportion of interactions within each network that had been previously reported by assembling comprehensive catalogs of human and mouse interactions, as described above. To gain insight into the likelihood that novel interactions detected by each method represented true positives, we withheld human interactions that had not been reported prior to 2017, and calculated the proportion of ‘novel’ interactions predicted from each human dataset that had been discovered in 2017, in a time-split cross-validation approach [20]. Interactions supported only by the PMIDs of previously published experiments analyzed here [13,15,32,33,35] were excluded from these analyses to eliminate circularity. To identify individual combinations of external genomic datasets that significantly increased the proportion of putative novel interactions that were subsequently discovered in our time-split cross-validation scheme, we compared each combination of external genomic features to the baseline networks recovered solely from dataset-derived features using a Brunner–Munzel test, followed by Bonferroni correction to control the family-wise error rate.

### Other outcomes

The ‘igraph’ package [64] in R was used to calculate the global clustering coefficient of each network. The number of publications referencing each protein was obtained from the ‘gene2pubmed’ file distributed by the NCBI [65].

### Statistical analysis

Spearman’s ρ was used to evaluate associations between number of genomic features and outcomes. To test for differences in functional connectivity between networks recovered with individual features and baseline networks, a two-sided Brunner–Munzel test was performed for each network, and the P-values were aggregated using Fisher’s method. To test for differences in proportion of novel interactions between individual feature and baseline networks, P-values from proportion tests performed for each network were aggregated using Fisher’s method. Differences between networks in bias towards highly studied proteins or global clustering coefficients were assessed using two-sided Brunner–Munzel tests.

## Supporting information

S1 Fig Novel interactions in PCP interactomes, with alternate time-split cross-validation. Interactions discovered in 2016 or later were withheld from the database of known interactions and used to estimate the proportion of true positives among putative novel interactions by time-split cross-validation.
(PDF)

S2 Fig Novel interactions in false discovery rate-controlled PCP interactomes. (A) Proportion of novel interactions within interaction networks at 50% precision recovered from co-migration data alone or supplemented with combinations of one to nine external genomic datasets. (B–C) Proportion of true positives among putative novel interactions by time-split cross-validation in false discovery rate-controlled networks, using interactions discovered in 2017 or later (B) and 2016 or later (C) to estimate the proportion of true positives.
(PDF)

S3 Fig Conclusions are robust to statistical framework used to predict interactions from PCP data. (A–E) Results obtained using support vector machines, instead of naive Bayes classifiers, to predict interactomes from 16 co-elution datasets. (A) Functional connectivity of PCP interactomes, predicted using machine-learning classifiers trained on raw co-migration data alone or supplemented with combinations of one to nine external genomic features (Spearman’s ρ = 0.46, P = 4.2 × 10^−68^). (B) Proportion of novel interactions in PCP interactomes recovered from co-migration data alone or supplemented with combinations of one to nine external genomic datasets (ρ = –0.33, P = 1.2 × 10^−34^). (C) Interactions discovered in 2017 only were withheld from the database of known interactions and used to estimate the proportion of true positives among putative novel interactions by time-split cross-validation (ρ = 0.082, P = 0.028). (D) Bias towards highly studied proteins in PCP interactomes, as quantified by Spearman correlation between protein degree and number of publications describing that protein, in interaction networks recovered from co-migration data alone, or supplemented with combinations of one to nine external genomic datasets (ρ = 0.29, P = 1.1 × 10^−26^). (E) Recovery of co-eluting complexes in PCP interactomes, as quantified by the global clustering coefficients of interactions recovered from co-migration data alone or supplemented with combinations of one to nine external genomic datasets (ρ = –0.085, P = 2.4 × 10^−3^).
(PDF)

S4 Fig Effect of individual genomic features on bias, protein complex recovery, and novelty. (A) Bias towards highly studied proteins in interaction networks recovered with individual external genomic features, compared to baseline. (B) Global clustering coefficients of interaction networks recovered with individual external genomic features, compared to baseline. (C–D) Proportion of true positives among putative novel interactions in interaction networks recovered with individual external genomic features, compared to baseline and estimated using time-split cross-validation.
(PDF)
