# Supplementary figures and images for: Genomic data integration systematically biases interactome mapping
Source: PLoS Comput Biol. 2018 Oct 17;14(10):e1006474. doi: 10.1371/journal.pcbi.1006474 (PMC6192561; doi:10.1371/journal.pcbi.1006474)

Dataset

- Kristensen
- Apoptosis
- Havugimana
- HeLa
- Kirkwood
- Wan

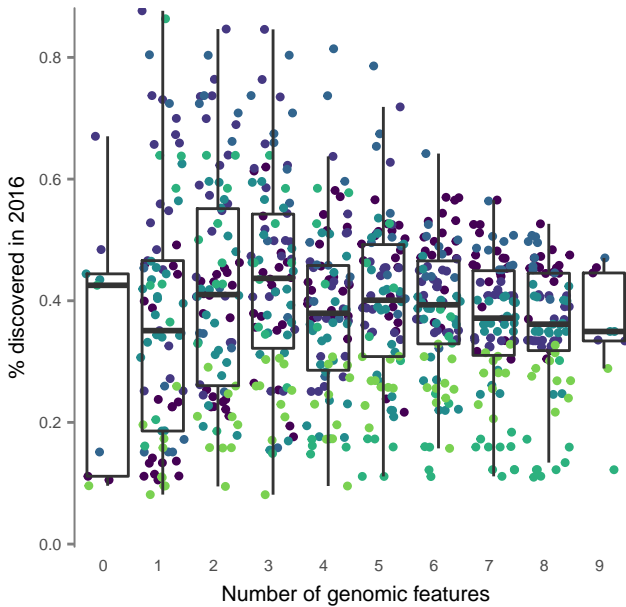

Supplement: S1 Fig — Interactions discovered in 2016 or later were withheld from the database of known interactions and used to estimate the proportion of true positives among putative novel interactions by time-split cross-validation. (PDF) [file pcbi.1006474.s001.pdf]

● Kristensen ● Apoptosis ● Havugimana  
● HeLa ● Kirkwood ● Tissues

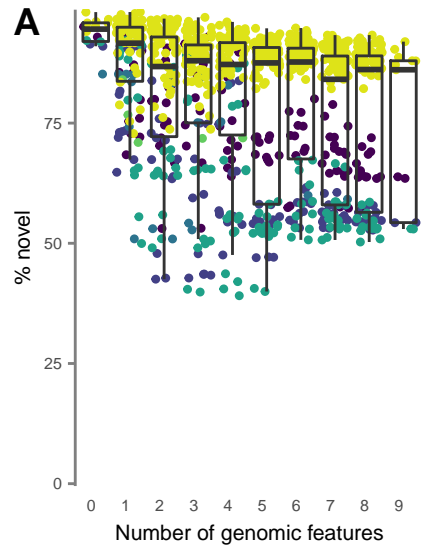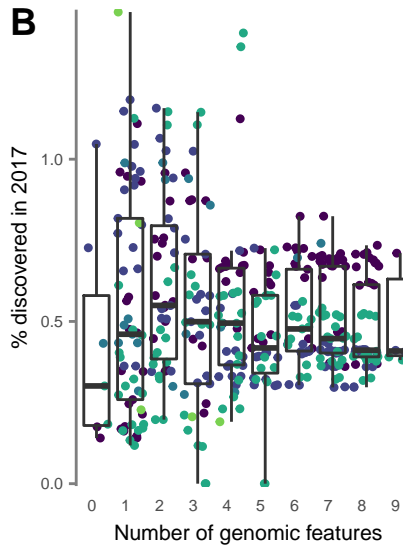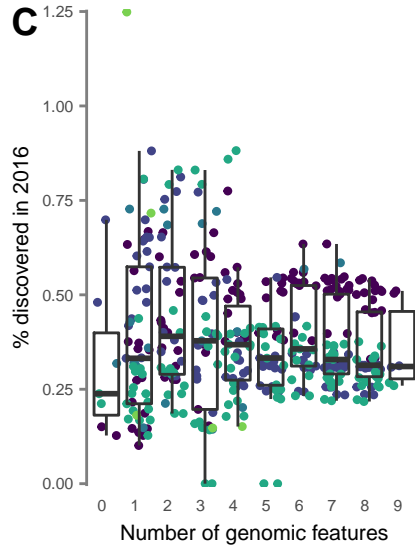

Supplement: S2 Fig — (A) Proportion of novel interactions within interaction networks at 50% precision recovered from co-migration data alone or supplemented with combinations of one to nine external genomic datasets. (B–C) Proportion of true positives among putative novel interactions by time-split cross-validation in false discovery rate-controlled networks, using interactions discovered in 2017 or later (B) and 2016 or later (C) to estimate the proportion of true positives. (PDF) [file pcbi.1006474.s002.pdf]

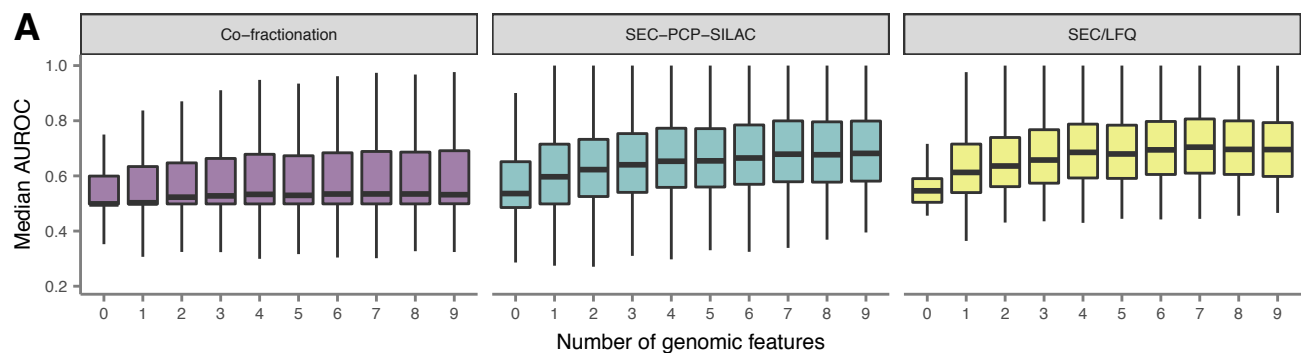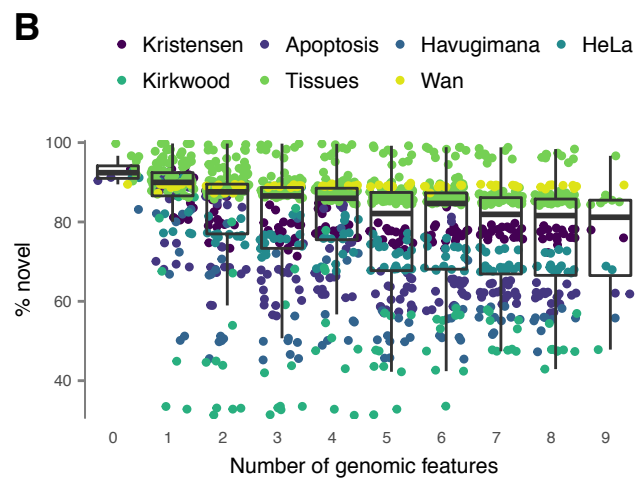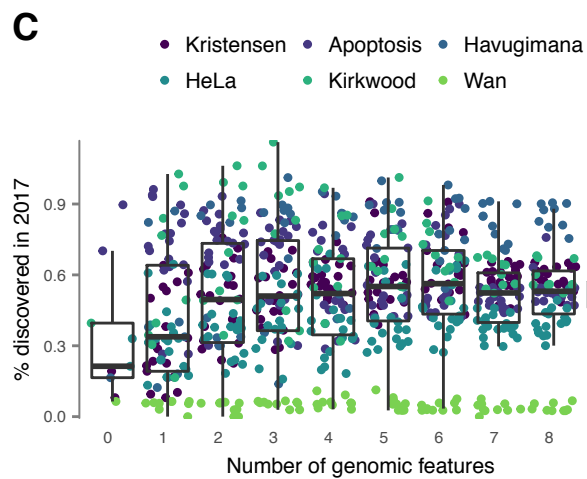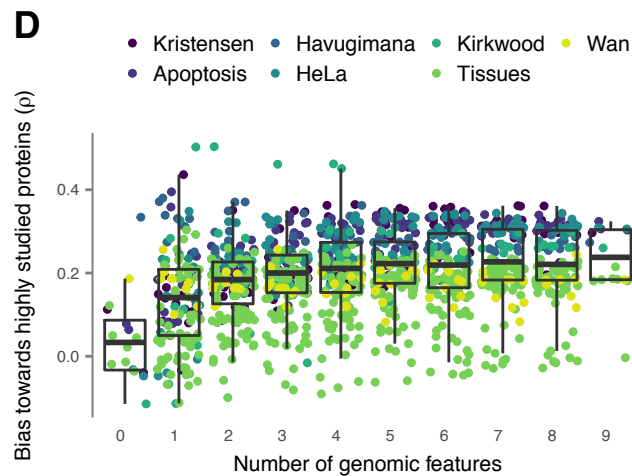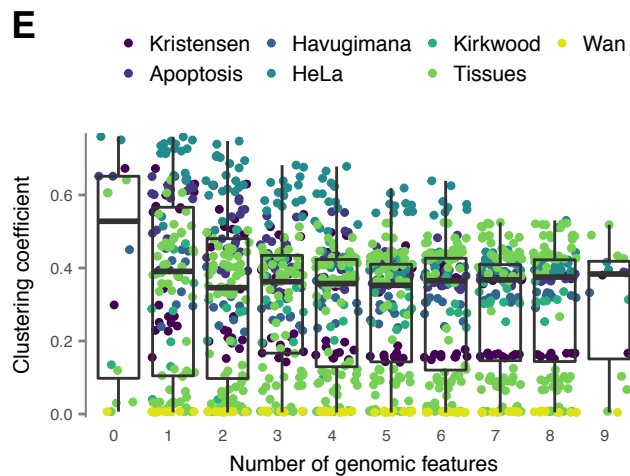

Supplement: S3 Fig — (A–E) Results obtained using support vector machines, instead of naive Bayes classifiers, to predict interactomes from 16 co-elution datasets. (A) Functional connectivity of PCP interactomes, predicted using machine-learning classifiers trained on raw co-migration data alone or supplemented with combinations of one to nine external genomic features (Spearman’s ρ = 0.46, P = 4.2 × 10^−68^). (B) Proportion of novel interactions in PCP interactomes recovered from co-migration data alone or supplemented with combinations of one to nine external genomic datasets (ρ = –0.33, P = 1.2 × 10^−34^). (C) Interactions discovered in 2017 only were withheld from the database of known interactions and used to estimate the proportion of true positives among putative novel interactions by time-split cross-validation (ρ = 0.082, P = 0.028). (D) Bias towards highly studied proteins in PCP interactomes, as quantified by Spearman correlation between protein degree and number of publications describing that protein, in interaction networks recovered from co-migration data alone, or supplemented with combinations of one to nine external genomic datasets (ρ = 0.29, P = 1.1 × 10^−26^). (E) Recovery of co-eluting complexes in PCP interactomes, as quantified by the global clustering coefficients of interactions recovered from co-migration data alone or supplemented with combinations of one to nine external genomic datasets (ρ = –0.085, P = 2.4 × 10^−3^). (PDF) [file pcbi.1006474.s003.pdf]

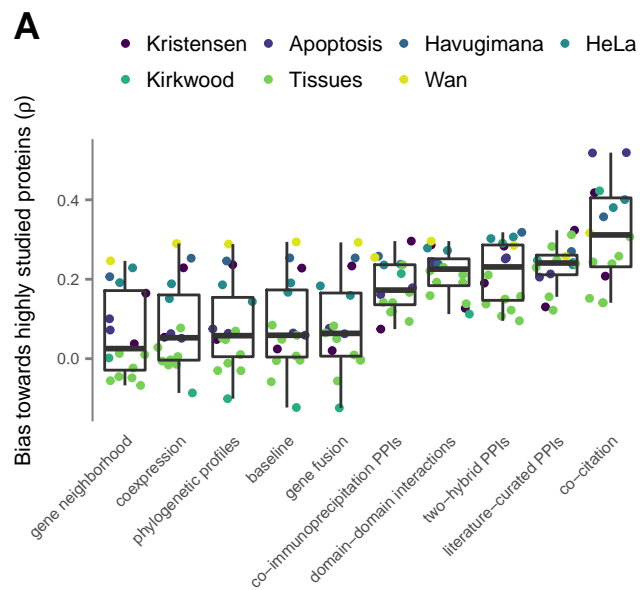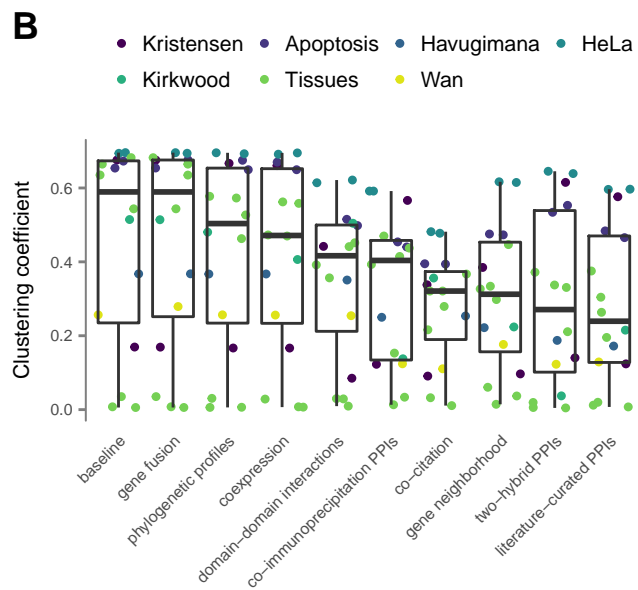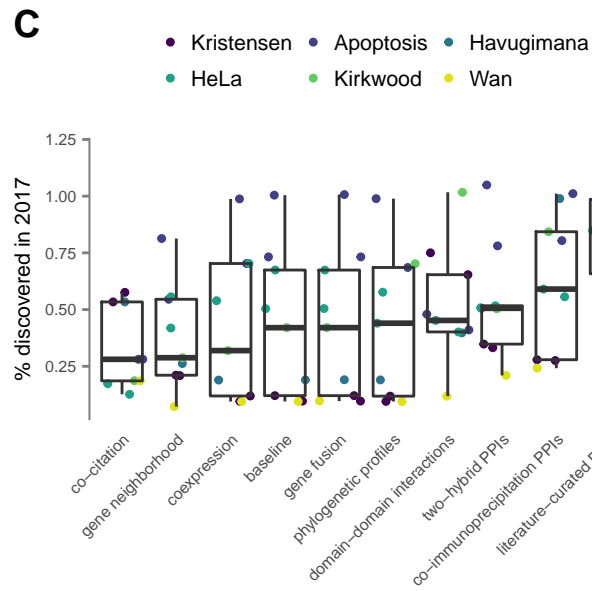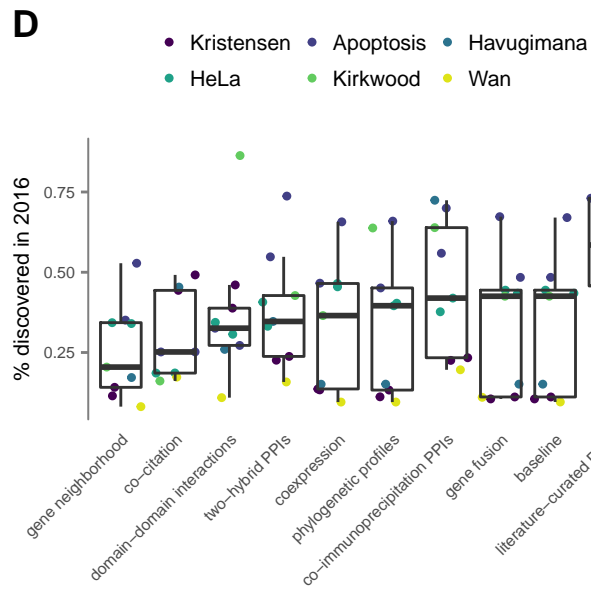

Supplement: S4 Fig — (A) Bias towards highly studied proteins in interaction networks recovered with individual external genomic features, compared to baseline. (B) Global clustering coefficients of interaction networks recovered with individual external genomic features, compared to baseline. (C–D) Proportion of true positives among putative novel interactions in interaction networks recovered with individual external genomic features, compared to baseline and estimated using time-split cross-validation. (PDF) [file pcbi.1006474.s004.pdf]
